# Prevalence and associated risk factors for mental health problems among young adults in Fiji Island during COVID-19: a cross-sectional study

**DOI:** 10.3389/fpubh.2023.1323635

**Published:** 2023-12-21

**Authors:** Mohammad G. M. Khan, Muhammad Mainuddin Patwary, Kabir A. Mamum, Aneesh A. Chand, Kaamil Edward, Kushal A. Prasad, Matthew H. E. M. Browning, Chaandvi Prasad, Faysal Kabir Shuvo

**Affiliations:** ^1^School of Information Technology Engineering Mathematics and Physics (STEMP), The University of the South Pacific, Suva, Fiji; ^2^Environment and Sustainability Research Initiative, Khulna, Bangladesh; ^3^Environmental Science Discipline, Life Science School, Khulna University, Khulna, Bangladesh; ^4^Department of Electronics, Instrumentation & Control Engineering, College of Engineering, Science & Technology, Fiji National University, Suva, Fiji; ^5^Department of Parks, Recreation, and Tourism Management, Clemson University, Clemson, SC, United States; ^6^College of Medicine, Nursing & Health Sciences, Fiji National University, Suva, Fiji; ^7^Swinburne University of Technology, Hawthorn, VIC, Australia

**Keywords:** mental health, COVID-19, pandemic, social restrictions, Fiji, Asia-Pacific

## Abstract

**Introduction:**

The COVID-19 pandemic has had a significant impact on mental health globally. To understand the impact of the pandemic on mental health in Fiji, this study aimed to investigate the prevalence of anxiety disorder and depression among the young adults.

**Method:**

An online survey was conducted to assess the prevalence of anxiety disorder and depression among the general population in Suva, Fiji during the COVID-19 pandemic. A total of 1,119 Fiji adults participated in the study. The study was conducted between May 20 to June 30, 2022, using a snowball sampling via social media platforms. The Generalized Anxiety Disorder (GAD-7) and Patient Health Questionnaire (PHQ-9) scales were used to measure anxiety and depression, respectively. The COVID-19 related stressors was evaluated using the adapted SARS stressors assessment. Univariate and multivariate logistic regression analysis was performed to determine the factors influencing mental health among respondents.

**Results:**

The result shows that a significant portion of individuals experienced each of the stressors, with the highest prevalence seen for hearing information about the severity of COVID-19. The prevalence of anxiety and depression was found to be 45% and 49%, respectively. Being female, having pre-existing illness and COVID-19 stressors were a risk factor to develop anxiety and depression. On the other hand, employed individuals and having high BMI was a protective factor against developing depression during COVID-19 lockdown.

**Conclusion:**

These findings highlight the importance of addressing the mental health needs of the Fijian population during the COVID-19 pandemic and beyond.

## 1 Introduction

The emergence of the Coronavirus disease (COVID-19) outbreak in late 2019 marked the beginning of a global health crisis that rapidly spread across the world (1). The virus was highly contagious, leading to a significant increase in cases and deaths outside of China in March 2020. The World Health Organization declared the outbreak an epidemic in January 2020, with over 200 countries and territories reporting cases (2). The COVID-19 can be transmitted through various routes, including direct transmission through physical contact, such as coughing, sneezing, and inhaling respiratory droplets from an infected person (3). Governments around the world had implemented various measures such as home confinement, quarantine for infected individuals, social distancing, and the use of personal protective equipment, such as face masks and gloves, in an attempt to control the spread of the virus (4). However, these containment strategies, including isolation and physical confinement, have reportedly had negative impacts on mental health (5). Frequent emotions and established risk factors for various mental health disorders, including anxiety, affective, and post-traumatic stress disorders, include frustration, loneliness, and worry about the future (6).

The COVID-19 pandemic has had a disproportionate impact on various groups, leading to increased mental health difficulties (7). In particular, healthcare workers are at higher risk of contracting the virus and experiencing heightened stress (8). Individuals with low income and precarious employment face job insecurity and live in overcrowded conditions, contributing to increased stress and depression. Marginalized communities experience systematic inequalities and limited access to healthcare and social support, amplifying the psychological impact of the crisis (9). The COVID-19 pandemic has also impacted children and school going students, placing them at risk of mental health difficulties from disrupting their learning process, and hindering their acquisition of knowledge, skills, and structured routines (10). Minority and ethnic groups, facing challenges such as key worker roles, overcrowded living conditions, poverty, and discrimination, are more susceptible to mental health issues. Individuals with pre-existing physical or mental health conditions are also at higher risk of mental health difficulties during COVID-19 because of worsening symptoms for individuals with pre-existing mental health conditions (7).

Fiji reported its first case of COVID-19 in Lautoka on 19 March 2020, and as of 1 June 2023, the country has had a total of 68,921 cases and 882 deaths, spanning across all divisions (11). The COVID-19 pandemic and the measures implemented to control its spread have disrupted daily routines, caused financial stress, and increased the risk of mental health problems in Fiji, including anxiety, depression, and substance abuse. The isolation and confinement resulting from the pandemic can worsen existing mental health issues, while limited access to mental health care services exacerbates the challenges due to cultural stigma, a shortage of trained professionals, and resource constraints. The economic consequences of the pandemic have also contributed to financial stress and further impacted mental health in Fiji (12). Ensuring the mental well-being of the population in Fiji will be crucial for the country’s overall recovery from the pandemic.

There is growing evidence that the COVID-19 has caused a substantial impact on mental health (13). The virus’s rapid and unprecedented transmission has fueled widespread fear, uncertainty, and anxiety, intensified by constant news updates and the perceived threat of infection (6, 14). The disruptions to routine life, including work, education, and daily activities, have resulted in psychosocial challenges, causing a loss of structure, purpose, and normalcy for many individuals. Financial hardships and economic uncertainties, compounded by job losses, have further heightened stress levels and distress (15). A meta-analysis that examined 68 studies from 19 countries during the pandemic found that approximately 33% of the general population experienced symptoms of anxiety, while 30% reported symptoms of depression (15). Early in the pandemic, a study in China noted alarming figures with 29% of the population experiencing anxiety and 37.1% grappling with depression (16). A broader international study covering 78 countries reported that 50% of individuals experienced moderate mental health effects due to COVID-19 lockdowns (17). The impact extends beyond high-income countries, with studies from 40 European countries reported a significant 17.80% prevalence of distress during the pandemic (18). Importantly, lower-income countries have not been spared, as evidenced by studies highlighting a higher prevalence of mental health issues during COVID-19 in low and lower-middle-income countries (19). The Asia-Pacific region, a diverse area with varied socio-economic landscapes, has consistently reported elevated levels of anxiety, depression, and stress during the pandemic (20). Specific attention has been drawn to Pacific Island countries, such as New Zealand, where a couple of studies have reported a higher proportion of psychological disturbances during COVID-19, shedding light on the unique challenges faced by these communities (21–23). Notably, there is a significant gap in studies on mental health during COVID-19 in Fiji Island underscores a broader issue of underrepresentation from certain regions, limiting the holistic understanding of the pandemic’s mental health impact.

The sharp rise in these mental health symptoms underscores the unique challenges and psychological distress caused by the pandemic. Studies reported that various factors associated with this increased susceptibility to psychological consequences during COVID-19 (15). The findings from recent studies indicate that certain demographic factors and risk factors are associated with a higher prevalence of mental health consequences. Specifically, females, younger age groups (24), individuals with lower socioeconomic status (SES) (25, 26), those residing in rural areas (27), people with preexisting illness (24), frequent alcohol-consumers (28), smokers (28), and individuals at higher risk of COVID-19 infection (29) and COVID-19 related stressors (30) are more likely to experience negative mental health.

While a substantial body of research has examined the impact of the COVID-19 pandemic on mental health globally (31–33), there is a significant dearth of systematic assessments specifically tailored to the Pacific Island context, particularly in Fiji. Much of the existing literature predominantly stems from studies conducted in diverse cultural and socioeconomic settings, potentially limiting its applicability to the unique circumstances of Pacific Island nations. The experiences of the researcher reveal a critical gap in understanding the mental health outcomes of individuals in Fiji during the ongoing pandemic. Unlike many developed nations, Fiji faces distinctive challenges, including limited mental health resources, unique cultural contexts, and vulnerability to external stressors (34). While one study conducted among 300 physical education and sports teachers in Fiji found that 50% of them were negatively affected by the pandemic (12), there was no research conducted on the broader population. Further, there is a pressing need for research that delves into the nuanced mental health experiences of the broader Fijian population during COVID-19, as existing interventions and findings may not be directly translatable to this specific cultural and regional context. Our study aims to address this gap by providing a focused examination of mental health outcomes in Fiji during the pandemic, contributing vital insights to the broader understanding of the pandemic’s impact on mental health in Pacific Island nations.

Nevertheless, it is important to recognize that negative psychological consequences were already evident in pacific region (35) and Fiji prior to the pandemic (34), and the COVID-19 situation has likely exacerbated these challenges. Fiji, like many other countries, has faced social and economic disruptions, health concerns, and increased stress levels due to the pandemic. The lack of systematic assessment of mental health outcomes among the general population in Fiji highlights the need for further research to understand the specific impact of the pandemic on mental health in the country. Therefore, this study investigated the mental health problems among different group of populations during the early stage of the COVID-19 pandemic in Fiji. We also aimed to examine the risk factors associated with developing these psychological problems, among general populations in Fiji.

## 2 Methods

### 2.1 Study design and data collection

An online survey was conducted to gather data on the impact of the COVID-19 pandemic on mental health in Suva, Fiji. The survey was conducted from May 20 to June 30, 2022, after the second wave of the lockdown. The target participants for the study were aged 18 or over, living in the Fiji during the survey. A total of 1,119 Fiji adults participated in the study. The sample was collected using the snowball sampling method, in which the survey was distributed through social networks such as Facebook, WhatsApp, LinkedIn, and Instagram. To design the questionnaire, we used Kobo Toolbox to create an online survey that could be accessed through a link. This allowed for easy data collection and visualization. The link for the survey was shared with the target group in two primary ways: (1) by emailing an invitation to participate to all students attending the University of the South Pacific and (2) by using field assistants to distribute the survey link to the target group.

### 2.2 Measurement instrument

#### 2.2.1 COVID-19 stress

The COVID-19 related stressors utilized in this study was adapted from the 10-item SARS stressors scale (36). It included seven questions related to COVID-19 infection, quarantine status, the severity of contagiousness, vacation and financial loss. Respondents answered each item as either yes or no, with a score of 1 or 0, respectively. The scores for all items were then summed to calculate an overall score, with higher scores indicating a greater amount of stress related to COVID-19.

#### 2.2.2 Mental health measures

To assess anxiety levels, we used the Generalized Anxiety Disorder 7-item (GAD-7) scale (37). This scale has excellent validity and reliability, with a Cronbach’s alpha coefficient of 0.911. Respondents indicate the frequency of symptoms over the past two weeks on a 0 (not at all) to 3 (almost every day) scale. A summary score is calculated by summing all items, with a range of 0 to 21. Respondents are categorized as having minimal/no anxiety (summary scores between 0–4), mild anxiety (5–9), moderate anxiety (10–14), or severe anxiety (15–21). In addition to these four levels of anxiety, we used a cutoff score of 9 or higher to identify clinical levels of generalized anxiety disorder (38).

To measure respondents’ levels of depression over the past two weeks, we used the Patient Health Questionnaire (PHQ-9). This well-validated tool has a Cronbach’s alpha coefficient of 0.89 and includes nine items that are rated on a 0 (not at all) to 3 (almost every day) scale (39). Scores are calculated by summing the items, with a range of 0 to 27. Scores of 0–4 indicate minimal to no depression, 5–9 mild depression, 10–14 moderate depression, and scores of 15–21 indicate severe depression (40). We used these four levels of depression, as well as a cutoff score of 10 or higher, to identify clinical levels of major depressive disorder (41).

#### 2.2.3 Risk factors

Sociodemographic variables including age, gender, level of education attained, living area, area of residence, current living status, occupations and monthly income were self-reported. Gender was determined by asking whether male or female or other. Age was used as a continuous variable. The education level of the participants was categorized into following groups including college, undergraduate and postgraduate level. Living region was assessed by identifying their region as Central, Northern, Eastern or Western. Residents’ characteristics were determined by their present residence, which was classified as urban or rural. Three questions were used to assess respondents’ living status, including whether they lived with or without family members or alone. Occupation were classified as unemployed, student, government job, private job, healthcare workers, teacher, business, daily labor worker, and housewife. Monthly income was classified as 0–2,000 or 2,001–4,000 or > 4,000 FJD.

In terms of health related variables, presence of a pre-existing illness, smoking habit, habit of drinking alcohol and kava, Body Mass index (BMI), self-reported health status and daily time spent for searching COVID-19 information were considered. Participants were asked if they have any long-standing illness or disability. BMI and daily time spent searching for COVID-19 information were recorded as continuous variables. Smoking and alcohol/kava consumption were determined by asking participants to indicate “yes” or “no”.

### 2.3 Data analysis

In our study, we used descriptive statistics to summarize the demographic characteristics of the respondents. We reported categorical data as percentages and continuous data as means and standard deviations. To check for data normality, we used the Shapiro–Wilk test. Since our data were not normally distributed, we used non-parametric tests to investigate the relationships between the respondents’ general characteristics and their mental health during the COVID-19 pandemic. To identify potential predictors of psychological outcomes, we conducted univariate (unadjusted) and multivariable (adjusted) logistic regression analysis, adjusting for sociodemographic factors. In the univariate analysis, we employed chi-squared tests or the Kruskal-Wallis test to assess the association between potential risk factors and psychological outcomes. Since we used cut-off values for outcome variables such as anxiety (≥10) and depression (≥10), we conducted multivariable logistic regression analysis after adjusting for sociodemographic, heath and COVID-19 stressors. We included only statistically significant predictors from the univariate analysis in the multivariable logistic regression models and calculated adjusted coefficients and their 95% confidence intervals for independent variables. We considered a two-tailed test with a significance level of *p* < 0.05 to be statistically significant. SPSS statistical software (version 26) was used to analyse the data.

### 2.4 Ethical approval consideration

The study followed the process of the Declaration of Helsinki and maintained the highest possible extent of ethical standards. The study included a clear description of the procedures followed to obtain informed consent from participants, including the purpose of the study, the voluntary nature of participation, and any necessary ethical approvals obtained from relevant institutional review boards or ethics committees. An electronic consent of participation was obtained from all the respondents before they took part in the study. The consent form is attached to the questionnaire. The study was approved by the ethical clearance committee of the School of Information Technology, Engineering, Mathematics and Physics (STEMP) Academic Unit Research Committee, University of South Pacific, Fiji.

## 3 Results

### 3.1 Demographic and health-related characteristics

Table 1 presents data on the sociodemographic and health-related characteristics of a group of respondents in Fiji. The majority of the respondents were female (59.8%), with a mean age of 26.01 years. In comparing our convenience sample to the known population parameters from the Fiji census, we observed slight variations in the age and gender distribution. The Fiji census data 2017 indicated that the median age of the population was 27.5 years, which means that half of Fiji’s population was below that age Additionally, the gender distribution was approximately 49% male and 51% female (42). There was a slight difference in the age distribution, with our sample having a slightly lower proportion (mean age: 26.01) compared to the population parameters. The gender distribution in our sample was also slightly different, with a higher percentage of females (59.8%).

**Table 1:** Demographic characteristic of the respondents (*N* = 1,119).

Features	*N*	Percentage (%), mean (SD)
**Gender**		
Male	445	39.8
Female	669	59.8
Non-binary or prefer not to say	5	0.4
**Age (years)**	1,119	26.01 (9.44)
**Marital status**		
Single	884	79.0
Married	215	19.2
Divorced	20	1.8
**Education**		
College or below	73	6.5
Undergraduate	660	59.0
Postgraduate or above	386	34.5
**Place of residence**		
Rural	203	18.1
Urban	916	81.9
**Occupation**		
Unemployed	51	4.6
Student	705	63.0
Employed	363	32.4
**Living status**		
Living alone	76	6.8
Living with family members	906	81.0
Living non-family members	137	12.2
**Monthly family income (FJD)**		
0–2,000	617	55.1
2,001–4,000	229	20.5
>4,000	273	24.4
**Having pre-existing illness**		
No	1,017	90.9
Yes	102	9.1
**Habit of smoking**		
No	971	86.8
Yes	148	13.2
**Habit of drinking alcohol**		
No	907	81.1
Yes	212	18.9
**Habit of drinking kava**		
No	928	82.9
Yes	191	17.1
**Body mass index (BMI)**	1,119	25.77 (5.9)
**Perceived health status**		
Poor	168	1.6
Fair	211	18.9
Good	478	42.7
Very good	245	21.9
Excellent	167	14.9
**Time spent for COVID-19 (h/day) information**	1,119	3.45 (3.17)

Most of the respondents were single (79.0%) and lived with their family members (81.0%). The majority of the respondents were students (63.0%), and enrolled in undergraduate education (59.0%). Most of the respondents lived in urban areas (81.9%) and had a monthly income of 0–2000 FJD (55.1%). In terms of health-related variables, only 9.1% of the respondents reported having a pre-existing illness. A small percentage of respondents reported engaging in smoking (13.2%) or drinking alcohol (17.4%) or drinking kava (17.1%). The mean body mass index (BMI) of the group was 25.77. In terms of perceived health status, 42.7% of the respondents reported being in good health, while 25.5% reported being in fair or poor health. The mean time spent searching for COVID-19 related information was 3.45 h per day.

### 3.2 COVID-19 stressors

**Figure 1:**
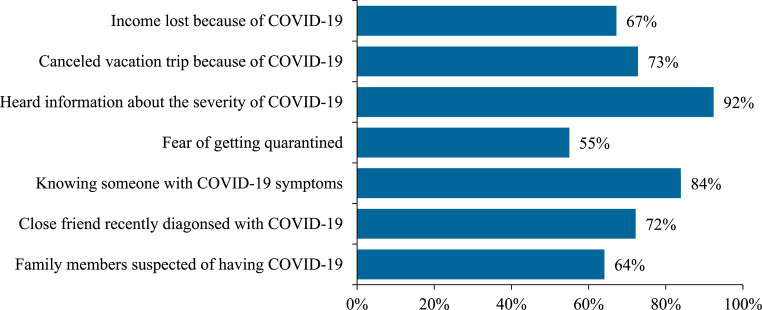
COVID-19 specific stresses among respondents (*N* = 1,119).

Figure 1 presents data on the prevalence of different COVID-19 specific stressors experienced by individuals. The figure includes data on seven different stressors: having family members suspected of having COVID-19, having a close friend recently diagnosed with COVID-19, knowing someone who has COVID-19 symptoms, fear of getting quarantined, hearing information about the severity of COVID-19, cancelling a vacation trip because of COVID-19, and experiencing income loss because of COVID-19.

The figure shows that a significant portion of individuals experienced each of the stressors, with the highest prevalence seen for hearing information about the severity of COVID-19 (92%), knowing someone who has COVID-19 symptoms (84%), cancelling a vacation trip because of COVID-19 (73%) and having a close friend recently diagnosed with COVID-19 (72%).

### 3.3 Overall prevalence of poor mental health

**Figure 2:**
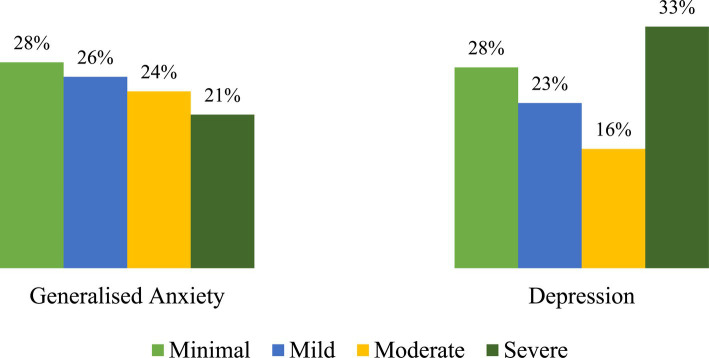
Overall prevalence of poor mental health (*N* = 1,119).

Figure 2 presents data on the prevalence of mental health issues among the general population in Fiji during the COVID-19 pandemic. For generalized anxiety, 28% of the general population had minimal levels of anxiety, 26% had mild levels, 24% had moderate levels, and 21% had severe levels. For depression, 28% reported minimal depression levels, 23% experienced mild depression, 16% had moderate depression levels, and 33% reported severe depression levels.

### 3.4 Prevalence of poor mental health by professions

Table 2 shows the prevalence of mental health among different groups of occupation. The prevalence of depression was significantly different between the unemployed (41.2%), students (47.2%), and employed (42.4%). No other differences between groups were detected.

**Table 2:** Prevalence of poor mental health by profession (*N* = 1,119).

Occupation	Anxiety (%)	95% CI	Depression (%)	95% CI
Unemployed	41.2	27.2–55.16	49.02	34.82–63.22
Student	47.2	43.54–50.93	54.33	50.64–58.01
Employed	42.4	37.32–47.53	49.6***	35.42–45.57

*** *p* < 0.001; anxiety cutoff score: ≥10; depression cutoff score: ≥10.

### 3.5 Factors influencing the prevalence of poor mental health

Table 3 presents the univariate and multivariate results for the risk factors for anxiety and depression. Gender, having pre-existing illness, and COVID-19 stress were significant predictors for experiencing anxiety. In particular, female respondents had a significantly increased risk of experiencing anxiety disorder (OR = 1.88 95% CI = 1.42–2.48, *p* < 0.001) than their counterparts. Respondents with a pre-existing illness were more likely to experience anxiety disorder (OR = 1.89, 95%CI = 1.19–3.01, *p* < 0.01). Further, respondents who had experienced higher COVID-19 stress during pandemic period were more likely to experience anxiety disorder (OR = 1.22, 95%CI = 1.13–1.32, *p* < 0.001).

**Table 3:** Influencing factors of mental health in Fiji during COVID-19.

Features (total *N*)	Anxiety disorder			Depression		
	*N* (%)	Univariate analysis, χ^2^	Multivariate analysis, OR (95% CI)	*N* (%)	Univariate analysis, *χ*^2^	Multivariate analysis, OR (95% CI)
**Gender**		20.77***			8.19**	
Male (445)	165 (37.1)		Ref.	198 (44.5)		Ref.
Female (669)	340 (50.8)		1.88 (1.42–2.48)***	353 (52.8)		1.35 (1.02–1.77)*
Non-binary or prefer not to say (5)	3 (60.0)		1.91 (0.29–12.36)	4 (80.0)		2.89 (0.30–27.55)
**Age (years)** (1119)	25.47 (±7.90)	3.95*	1.00 (0.98–1.02)	25.17 (±8.01)	12.82***	1.00 (0.97–1.01)
**Marital status**		5.37*			8.18**	
Single (884)	418 (47.3)		Ref.	459 (51.9)		Ref.
Married (215)	78 (36.3)		0.70 (0.47–1.05)	83 (38.6)		0.77 (0.51–1.15)
Divorced (20)	12 (60.0)		2.08 (0.78–5.56)	13 (65.0)		2.35 (0.83–6.57)
**Education**		0.026			0.002	
College or below (73)	32 (43.8)		Ref.	29 (39.7)		Ref.
Undergraduate (660)	303 (45.9)		1.10 (0.62–1.93)	340 (51.5)		1.70 (0.95–3.03)
Postgraduate or above (386)	173 (44.8)		1.28 (0.71–2.32)	186 (48.2)		2.30 (1.24–4.24)
**Place of residence**		2.03			0.068	
Rural (203)	83 (40.9)		Ref.	99 (48.8)		Ref.
Urban (916)	425 (46.4)		1.23 (0.87–1.74)	456 (49.8)		0.91 (0.64–1.28)
**Occupation**		1.20			13.05***	
Unemployed (51)	21 (41.2)		Ref.	25 (49.0)		Ref.
Student (705)	333 (47.2)		1.12 (0.60–2.12)	383 (54.3)		1.04 (0.55–1.96)
Employed (363)	154 (42.4)		0.91 (0.41–2.02)	25 (36.2)		0.43 (0.19–0.97)*
**Living status**		2.19			2.35	
Living alone (76)	37 (48.7)		Ref.	40 (52.6)		Ref.
Living with family members (906)	396 (43.7)		0.76 (0.45–1.28)	434 (47.9)		0.66 (0.38–1.12)
Living non-family members (137)	75 (54.7)		1.14 (0.62–2.12)	81 (59.1)		0.97 (0.51–1.80)
**Monthly family income (FJD)**		9.22**			3.13	
0–2,000 (617)	306 (49.6)		Ref.	319 (51.7)		Ref.
2,001–4,000 (229)	93 (40.6)		0.70 (0.50–0.97)	113 (49.3)		1.05 (0.75–1.46)
>4,000 (273)	109 (39.9)		0.72 (0.52–0.99)	123 (45.1)		0.81 (0.59–1.11)
**Having pre-existing illness**		3.63***			14.61***	
No (1017)	444 (43.7)		Ref.	486 (47.8)		Ref.
Yes (102)	64 (62.7)		1.89 (1.19–3.01)**	69 (67.6)		1.97 (1.21–3.19)
**Habit of smoking**		1.45			2.86	
No (971)	434 (44.7)		Ref.	472 (48.6)		Ref.
Yes (148)	74 (50.0)		1.31 (0.86–1.99)	83 (56.1)		1.46 (0.95–2.23)
**Habit of drinking alcohol**		0.53			0.34	
No (907)	407 (44.9)		Ref.	446 (49.2)		Ref.
Yes (212)	101 (47.6)		1.03 (0.71–1.50)	109 (51.4)		1.08 (0.73–1.57)
**Habit of drinking kava**		0.07			1.14	
No (928)	423 (45.6)		Ref.	467 (50.3)		Ref.
Yes (191)	85 (44.5)		0.87 (0.58–1.30)	88 (46.1)		0.70 (0.46–1.05)
**Body mass index (BMI)** (1119)	25.71 (±5.91)	0.24	0.99 (0.97–1.02)	25.47 (±5.98)	4.00*	0.97 (0.95–0.98)*
**Perceived health status**		19.04***			32.65***	
Poor (168)	12 (66.7)		Ref.	12 (66.7)		Ref.
Fair (211)	117 (55.5)		0.75 (0.25–2.26)	127 (60.2)		1.14 (0.38–3.40)
Good (478)	219 (45.8)		0.54 (0.18–1.59)	254 (53.1)		0.80 (0.27–2.35)
Very good (245)	101 (41.2)		0.49 (0.16–1.48)	103 (42.0)		0.54 (0.18–1.63)
Excellent (167)	59 (35.3)		0.41 (0.13–1.27)	59 (35.3)		0.42 (0.13–1.28)
**Time spent for COVID-19 (h/day) information** (1119)	3.67 (±3.17)	7.73**	1.04 (1.00–1.08)	3.48 (±3.08)	0.99	1.00 (0.95–1.04)
**COVID-19 stress** (1119)	5.40 (±1.55)	29.46***	1.22 (1.13–1.32)***	5.34 (±1.57)	23.65***	1.20 (1.12–1.30)***

*p < 0.05, **p < 0.01, ***p < 0.001; bold are significant value; anxiety cutoff score: ≥10; depression cutoff score: ≥10.

Similarly, gender, occupation, BMI, and COVID-19 stress were significant factors related to depression. Female respondents had a greater risk of experiencing depression (OR = 1.35 95% CI = 1.02–1.77, *p* < 0.05) than their counterparts. Respondents who experienced higher COVID-19 stress were more prone to experience depression (OR = 1.23, 95%CI = 1.12–1.30, *p* < 0.001). However, employed individuals (OR = 0.43 95%CI = 0.19–0.97, *p* < 0.05), and those had a high BMI (OR = 0.97, 95%CI = 0.95–0.98, *p* < 0.5) were less likely to experience depression (Table 3).

## 4 Discussion

### 4.1 Summary of main findings

In Fiji, mental health issues have been a growing concern for many years, with limited resources and access to mental health services for the general population (43). The COVID-19 pandemic exacerbated this issue, with a significant portion of the population experiencing mental health challenges such as anxiety and depression. The pandemic also resulted in unprecedented changes in daily life, including lockdowns, social distancing, and travel restrictions. It not only posed a significant threat to physical health but also had a substantial impact on mental health. Addressing mental health impacts during lockdown periods in Fiji was limited. The current study of 1,119 Fiji young adults determined prevalence and risk factors of poor mental health during the pandemic.

Our findings suggest that a significant portion of the young adults in Fiji experienced mental health issues, with higher levels of severity seen for depression than generalized anxiety. Given the scarcity of existing national study on the impact of COVID-19 on mental health in Fiji, we have compared our study findings with other countries. A similar finding was observed in young adults in New Zealand during the COVID-19 outbreak (44). Studies from around the world have consistently reported an increase in mental health issues including depression, anxiety, and stress during the pandemic due to various stressors such as isolation, fear of infection, financial difficulties, and loss of loved ones (45, 46). Interestingly, we found that students consistently showed the highest rates of depression, followed closely by the unemployed and then the employed. Previous studies also reported that students were highly susceptible to developing mental health than working professionals during COVID-19 (47). Students faced significant disruptions in their education during the COVID-19 pandemic, including the shift to online learning, which often introduced new challenges and stressors (48). The uncertainty of the academic environment and the need to adapt to remote learning can contribute to increased anxiety and depression among students (49). Students often faced uncertainty about the future, including concerns about job prospects, internships, or the continuation of their education that could lead to depression (50). Our study also suggests that COVID-19 had a significant impact on individuals and caused a range of related stressors among the adult population in Fiji. Similar findings were observed in other developing countries like India (51) and Bangladesh (47). It’s important to consider the potential impact of these stressors on mental health and to address the needs of individuals who have experienced them.

Our findings suggest that female respondents had a significantly increased risk of experiencing anxiety disorder and depression than their counterparts. This finding is in line with previous research that has shown that women are more likely to experience anxiety disorders and depression than men (52–54). Such gender differences may correspond to women being more affected by the social and economic consequences of the pandemic than men on average (55). For instance, school closures and family members becoming unwell may result in additional caregiving responsibilities for women. Women are also more likely to be financially disadvantaged during the pandemic due to lower salaries, less savings, and less secure employment than men (56, 57). As a result, women may be more vulnerable to financial stress and insecurity, which can increase the risk of developing mental health disorders (58). Furthermore, the prevalence of domestic violence has increased during periods of lockdown and stay-at-home orders, with women being more likely to be victims of such violence (59). The pandemic has exacerbated existing gender inequalities and increased the burden of caregiving and household responsibilities, which may contribute to the higher prevalence of mental health disorders among women (57). Other reasons could be biological mechanisms such as that there are hormonal differences between males and females that affect the way they respond to stress. Research has shown that women tend to have higher levels of stress hormones like cortisol and may be more sensitive to the effects of these hormones on their bodies and brains (60).

Having a pre-existing illness was a risk factor to develop anxiety disorder during the pandemic in Fiji. These findings were consistent with previous studies (61–63). People with pre-existing health conditions may be at higher risk of developing severe COVID-19 symptoms, which can increase anxiety and fear about their health and wellbeing. They may worry about the potential consequences of contracting COVID-19 and the impact it could have on their health and ability to manage their existing illness (64). Further, people with chronic illnesses may have more limited access to healthcare services during the pandemic, which can lead to increased anxiety about their ability to manage their illness and access the care they need. The pandemic has disrupted healthcare systems and forced many people to delay or forego medical appointments, which can exacerbate feelings of uncertainty and anxiety (65).

Our study suggests that higher COVID-19 stress was a risk factor to develop anxiety and depression. In this study, having family members or a close friend or knowing someone suspected of COVID-19, hearing information about the severity of COVID-19, cancelling a vacation trip because of COVID-19, and experiencing income loss because of COVID-19 was the major COVID-19 stress that elevated respondents’ risk of anxiety and depression. These findings are consistent with earlier studies (66, 67). Unemployment, for instance, can lead to financial insecurity and a sense of loss of control over one’s life, both of which are known risk factors for depression and anxiety (68). The death of a loved one or friend due to COVID-19 can also cause intense grief and distress, leading to the development of these mental health conditions. Receiving a positive COVID-19 diagnosis can also cause fear and uncertainty about one’s health and the health of others, which can lead to anxiety symptoms. Further, individuals may have been worried about their family members getting infected by the virus. This was especially true for working professionals who had to return to their workplaces during the pandemic, such as healthcare workers who were more susceptible to exposure and could transmit the virus to their families (69). Research found that participants whose family members worked in healthcare were 44% more likely to develop mental illness (70). The perceived risk of contracting or transmitting the virus to family members contributed to increased stress and anxiety among workers.

Interestingly, our findings found that employed individuals were protective against depression during lockdowns. Employed individuals typically have a source of income and financial stability. This financial security can reduce the stress associated with economic uncertainty, which is a common trigger for depression, especially during economic downturns (71, 72). One study conducted in Turkey found that state employees experienced lower levels of anxiety and depression compared to those in private sectors (73). Further, employees in Fiji may have had access to better resources and support systems during the pandemic, which could have contributed to their better mental health outcomes. For example, government employees may have had access to mental health services through their employee assistance programs, as well as job protections and financial support during the pandemic. However, further research is needed to confirm this finding and explore potential explanations for this relationship.

High BMI was associated with less depression in our study. High BMI being considered a protective factor might seem counterintuitive, especially given the common perception of high BMI as an adverse health outcome. It’s crucial to note that the relationship between BMI and health outcomes can be complex and context-dependent. While high BMI is generally associated with increased health risks (74), in certain populations or contexts, it may indeed be linked to better health outcomes. One theoretical rationale for high BMI being perceived as protective, particularly in some low-income populations, could be related to the “obesity paradox.” This phenomenon suggests that, in certain conditions such as chronic diseases or in older age groups, individuals with a higher BMI might have a survival advantage compared to those with a lower BMI. This paradox has been observed in a previous study where overweight groups exhibited the lowest prevalence of depression (75). It is often attributed to factors like better nutritional reserves, increased energy stores, and potential protective effects in the face of certain health challenges. Further, it is well established that physical activity is beneficial for mental health, and individuals with a higher BMI may be more likely to involve in weight-gain protective behavior (76). Physical activity has also been shown to have a positive impact on the immune system, which may be particularly relevant during a pandemic.

### 4.2 Implications of the study

Our study findings have important implications. The high prevalence rates of anxiety disorder and depression underscore the critical need for strengthened mental health support services in Fiji, especially during public health crises like the COVID-19 pandemic. Investing in accessible and culturally sensitive mental health resources can aid in addressing the immediate mental health needs of the population (77). Our study findings underscore the need for targeted public health interventions to address mental health challenges in Fiji, particularly among vulnerable groups such as students, females, and individuals with pre-existing illnesses. Implementing support programs that address the specific needs and challenges faced by these populations can contribute to more effective mental health outcomes. Community-based mental health programs should be considered to foster a supportive environment. These programs can engage community leaders, local organizations, and individuals to create a network of mental health support, reducing stigma and promoting open conversations about mental well-being (78). Understanding the generational impact of mental health, policymakers should focus on long-term resilience-building measures. Incorporating mental health education in schools, workplaces, and community settings can contribute to a more resilient and mentally healthy future population (79). However, our findings should be interpreted with caution. Generalizing to rural populations or those with different socio-economic and cultural backgrounds should be approached with caution. Fiji’s diversity may result in varied responses to stressors and different prevalence rates. Extrapolating findings to populations outside Fiji, especially in vastly different cultural and socio-economic contexts, may not be appropriate. The uniqueness of Fiji’s circumstances necessitates careful consideration when applying these findings to dissimilar settings.

### 4.3 Limitations of the study

The participants were recruited through convenience sampling, which may limit the generalizability of the findings to the larger population in Fiji. The study participants were also recruited through social media, which may result in a self-selection bias. The data collected in this study was based on self-reported measures, which may be subject to recall bias, social desirability bias, and other sources of response bias. The study used a cross-sectional design, which limits the ability to draw causal inferences between the COVID-19 pandemic and mental health outcomes. Longitudinal studies would be necessary to assess the temporal relationships between exposure to the pandemic and mental health outcomes. Further, we adapted the SARS-10 scale for assessing COVID-19 stressors was influenced by the historical context and the imperative for a validated instrument at the onset of our study. During that period, there was no well-established COVID-19 stressor scale. Further, the SARS-10 scale stood as a well-established and widely recognized tool for evaluating stressors related to infectious disease outbreaks. The study relied on self-reported symptoms of depression and anxiety, rather than clinical diagnoses made by healthcare professionals. Further, the study did not collect information on COVID-19 exposure, such as whether participants had contracted the virus or had close contacts who did, which may be an important factor in understanding mental health outcomes during the pandemic.

## 5 Conclusion

In conclusion, it is imperative to delve deeper into the implications of the study findings and consider the broader context for mental health interventions in Fiji. The substantial prevalence rates of anxiety disorder and depression uncovered in this study underscore the urgent need for targeted mental health interventions tailored to the unique challenges faced by the general population in Suva, Fiji. The prevalence rates, particularly the high prevalence related to hearing information about the severity of COVID-19, emphasize the pervasive impact of pandemic-related stressors on mental well-being. Moreover, the identified risk factors, such as being female, having a pre-existing illness, and exposure to COVID-19 stressors, provide critical insights into the specific demographic and contextual elements that amplify the vulnerability to anxiety and depression. These risk factors should guide the development of interventions that address the distinct needs of these at-risk groups. Conversely, the protective factors identified, including employment status and higher BMI, present valuable opportunities for targeted mental health support strategies. Recognizing the potential resilience conferred by employment and certain health characteristics can inform interventions designed to bolster mental well-being in these specific segments of the population. These findings serve as a foundation for evidence-based mental health initiatives in Fiji during future public health crises. The identified risk and protective factors should be integrated into public health strategies, with a focus on proactive and accessible mental health support systems. Policymakers and healthcare professionals can leverage this knowledge to implement interventions that not only address the current challenges but also fortify mental health resilience for the future.

## Data availability statement

The raw data supporting the conclusions of this article will be made available by the authors, without undue reservation.

## Ethics statement

The studies involving humans were approved by School of Information Technology, Engineering, Mathematics and Physics (STEMP) Academic Unit Research Committee, University of South Pacific, Fiji. The studies were conducted in accordance with the local legislation and institutional requirements. Written informed consent for participation in this study was provided by the participants’ legal guardians/next of kin.

## Author contributions

MK: Conceptualization, Funding acquisition, Methodology, Writing – review & editing. MP: Conceptualization, Data curation, Formal analysis, Methodology, Writing – original draft, Writing – review & editing. KM: Conceptualization, Funding acquisition, Investigation, Methodology, Project administration, Supervision, Writing – review & editing. AC: Conceptualization, Data curation, Methodology, Writing – review & editing. KE: Conceptualization, Data curation, Writing – review & editing. KP: Conceptualization, Data curation, Writing – review & editing. MB: Conceptualization, Methodology, Writing – review & editing. CP: Conceptualization, Methodology, Writing – review & editing. FS: Conceptualization, Methodology, Writing – review & editing.
